# cGMP-Phosphodiesterase Inhibition Enhances Photic Responses and Synchronization of the Biological Circadian Clock in Rodents

**DOI:** 10.1371/journal.pone.0037121

**Published:** 2012-05-10

**Authors:** Santiago A. Plano, Patricia V. Agostino, Horacio O. de la Iglesia, Diego A. Golombek

**Affiliations:** 1 Departamento de Ciencia y Tecnología, Universidad Nacional de Quilmes/CONICET, Buenos Aires, Argentina; 2 Department of Biology, University of Washington, Seattle, Washington, United States of America; Morehouse School of Medicine, United States of America

## Abstract

The master circadian clock in mammals is located in the hypothalamic suprachiasmatic nuclei (SCN) and is synchronized by several environmental stimuli, mainly the light-dark (LD) cycle. Light pulses in the late subjective night induce phase advances in locomotor circadian rhythms and the expression of clock genes (such as *Per1-2*). The mechanism responsible for light-induced phase advances involves the activation of guanylyl cyclase (GC), cGMP and its related protein kinase (PKG). Pharmacological manipulation of cGMP by phosphodiesterase (PDE) inhibition (e.g., sildenafil) increases low-intensity light-induced circadian responses, which could reflect the ability of the cGMP-dependent pathway to directly affect the photic sensitivity of the master circadian clock within the SCN. Indeed, sildenafil is also able to increase the phase-shifting effect of saturating (1200 lux) light pulses leading to phase advances of about 9 hours, as well as in C57 a mouse strain that shows reduced phase advances. In addition, sildenafil was effective in both male and female hamsters, as well as after oral administration. Other PDE inhibitors (such as vardenafil and tadalafil) also increased light-induced phase advances of locomotor activity rhythms and accelerated reentrainment after a phase advance in the LD cycle. Pharmacological inhibition of the main downstream target of cGMP, PKG, blocked light-induced expression of *Per1*. Our results indicate that the cGMP-dependent pathway can directly modulate the light-induced expression of clock-genes within the SCN and the magnitude of light-induced phase advances of overt rhythms, and provide promising tools to design treatments for human circadian disruptions.

## Introduction

In mammals, the master circadian clock resides in the hypothalamic suprachiasmatic nuclei (SCN), which is able to adjust its parameters with a variety of environmental signals. The light-dark (LD) cycle is the principal synchronizer of the biological clock [1], [2]; photic information reaches the SCN through the retinohypothalamic tract (RHT) by means of a glutamatergic signal, resulting in phase shifts of biological rhythms [3]–[6]. Exposure to light pulses (LPs) at night synchronizes the clock by inducing phase delays during the early night and phase advances during the late night [7], [8]. In hamsters, responses to light during the subjective night are mediated through a common signaling pathway involving glutamate, Ca^2+^, CaMKII and neuronal nitric oxide synthase (nNOS), which couple photic stimulation to the transcriptional activation of clock genes [9], [10]. However, downstream of nitric oxide these pathways bifurcate [11]. During the late night, the activation of a cGMP-dependent pathway —including the activation of guanylyl cyclase (GC)— and the activation of a cGMP dependent protein kinase (PKG) is involved in phase advances [12]–[14]. Pharmacological manipulation of cGMP by inhibition of its degradation pathway (i.e., by blocking cGMP-phosphodiesterase (PDE) should therefore affect photic entrainment and resynchronization.

cGMP-specific phosphodiesterase (PDE) inhibitors, which prevent the hydrolysis of cGMP, allow the accumulation of cGMP in the cells. Since several PDE families have been described [15], it is important to consider the specificity of such inhibitors. In particular, sildenafil directly inhibits the breakdown of cellular cGMP by PDE5 [16] and thereby prolongs and enhances the effects of NO/cGMP. In a previous study we demonstrated the presence of PDE5 in the hamster SCN, and the ability of sildenafil to both accelerate circadian resynchronization after an abrupt 6-h advance of the LD cycle and to enhance advances of rhythmic locomotor activity in response to a single LP during the subjective night [17].

We hypothesized that the enhancement of phase advances by PDE inhibitors could reflect the ability of the cGMP-dependent pathway to directly affect the photic sensitivity of the master clock within the SCN. We tested this hypothesis by assessing the ability of sildenafil to enhance phase advances in response to saturating levels of light in hamsters, as well as in a mouse strain that shows reduced phase advances. We also tested the ability of pharmacological inhibition of the main downstream target of cGMP, PKG, to block light-induced expression of *Per1*. Because of the potential utility of PDE inhibitors as pharmacological agents to accelerate entrainment to advances of the LD cycle, we tested the ability of PDE inhibitors other than sildenafil to enhance light induced phase advances, as well as the ability of sildenafil to enhance light-induced advances after intraperitoneal (ip.) injections in female hamsters and oral administration in male hamsters.

The aim of the present work was to expand our previous findings in the role of PDE in the circadian system [17]. In particular we aimed to determine whether PDE inhibition affects the photic sensitivity of the mammalian circadian clock. We used additional approaches to this question, expanding the drugs to be tested, using a different species (i.e. mouse), testing several light intensities and also including male and female hamsters. All these were combined with *in situ* hybridization and immunocytochemistry to assess the state of the molecular clock. Taken together, our results suggest that the cGMP-dependent pathway can directly modulate the light-induced expression of clock-genes within the SCN and the magnitude of light-induced phase advances of its master circadian oscillator.

## Results

**Figure 1:**
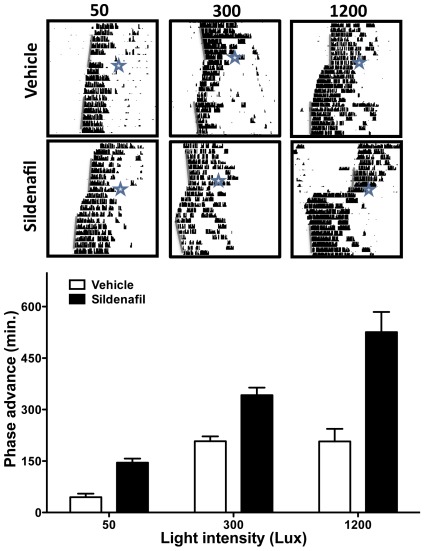
Sildenafil administration increases light-induced phase advances. In control animals a 300 lux light pulse induced a significantly higher phase advance than a 50 lux light pulse, while there was no difference between the 1200 lux and 300 lux groups. In contrast, the analysis of sildenafil treatment shows significant differences within the three groups (see text for details). All data represented as mean (± SD). Representative actograms (top) are presented for vehicle or drug treatment; time of injection and LP are indicated by a star, a grey bar shows estimated onset.

In a previous study we demonstrated that inhibition of PDE5 by sildenafil increases a 50-lux light-induced phase advance but has no effect when administered by itself in the absence of a LP, and accelerates circadian reentrainment to a 6-h advance of the LD cycle [17]. Our results suggest a possible role for PDEs in modulating the sensitivity of the circadian system to photic stimulation. Whereas low-intensity LPs have no significant effect on the phase of circadian locomotor activity, LPs above a given intensity threshold saturate the phase advance responses [18]. We therefore tested the hypothesis that sildenafil could increase the ceiling effect for light stimulation. We assessed the effect of sildenafil or vehicle on sub-saturating (50 lux) saturating (300 lux) and supra-saturating (1200 lux) light-induced phase advances (Fig. 1). A two-way ANOVA yielded significant effects of treatment and light intensity (p<0.0001), as well as for their interaction (p = 0.0036). This interaction was the result of the non-additive effect of light intensity in control animals. A 50 lux LP 30 min after vehicle administration induced a significantly lower phase advance than a 300 lux and a 1200 lux pulse, but the advances did not differ between 300 and 1200-lux treated animals (50 lux: 49±11.44 min n = 6; 300 lux: 204±22 min n = 6; 1200 lux: 234±42.3 min n = 8; p<0.0002, one-way ANOVA, followed by Tukey's test, 50 vs. 300 lux, p<0.0005; 300 vs. 1200 lux, n.s.; 50 vs. 1200 lux, p<0.0005). In contrast, sildenafil increased the phase advance induced by a 1200-lux pulse when compared to both a 50-lux and a 300-lux pulse (50 lux: 144.8±14.68 min n = 6; 300 lux: 336±67 min n = 6; 1200 lux: 561±57.7 min n = 8, p<0.0001, one-way ANOVA, Tukey's test: 50 vs. 300 lux, p<0.005; 300 vs. 1200 lux, p<0.005; for 50 vs. 1200 lux, p<0.0005). In addition, sildenafil reduced the number of transient days after the 1200 lux and the 300 lux LP until a new stable phase was achieved (2.57±0.42 days vs. 5.33±0.42 days in control animals, p<0.001 and 5.67±0.49 days in sildenafil-treated vs. 7.16±0.30 days in control animals, p = 0.027 respectively, Student's t-test) (data not shown).

In order to discard direct retinal effects of this PDE inhibitor, we performed electroretinographic recordings following ip. administration of 3.5 mg/kg sildenafil. No significant changes were found for amplitude nor latency of the *a* (amplitude: controls 36±5 µV vs sildenafil 41±4 µV; latency: controls 28±0.7 msec vs sildenafil 29±1.2 msec) nor the *b* wave of the ERG (amplitude: controls 92±8 µV vs sildenafil 102±7 µV; latency: controls 53±2 msec vs sildenafil 57±2 msec) after sildenafil administration (Figure S1).

Taken together, our results demonstrate that sildenafil (i.e., PDE inhibition) affects the light-input threshold for circadian phase shifts. Furthermore, the intensity-response curve in sildenafil-treated animals indicates a linear relationship between light intensity and phase advances, with no plateau achieved with stimulations of up to 1200 lux. In other words, PDE inhibition has the ability to enhance light-induced phase advances beyond the saturation point for vehicle-treated animals.

**Figure 2:**
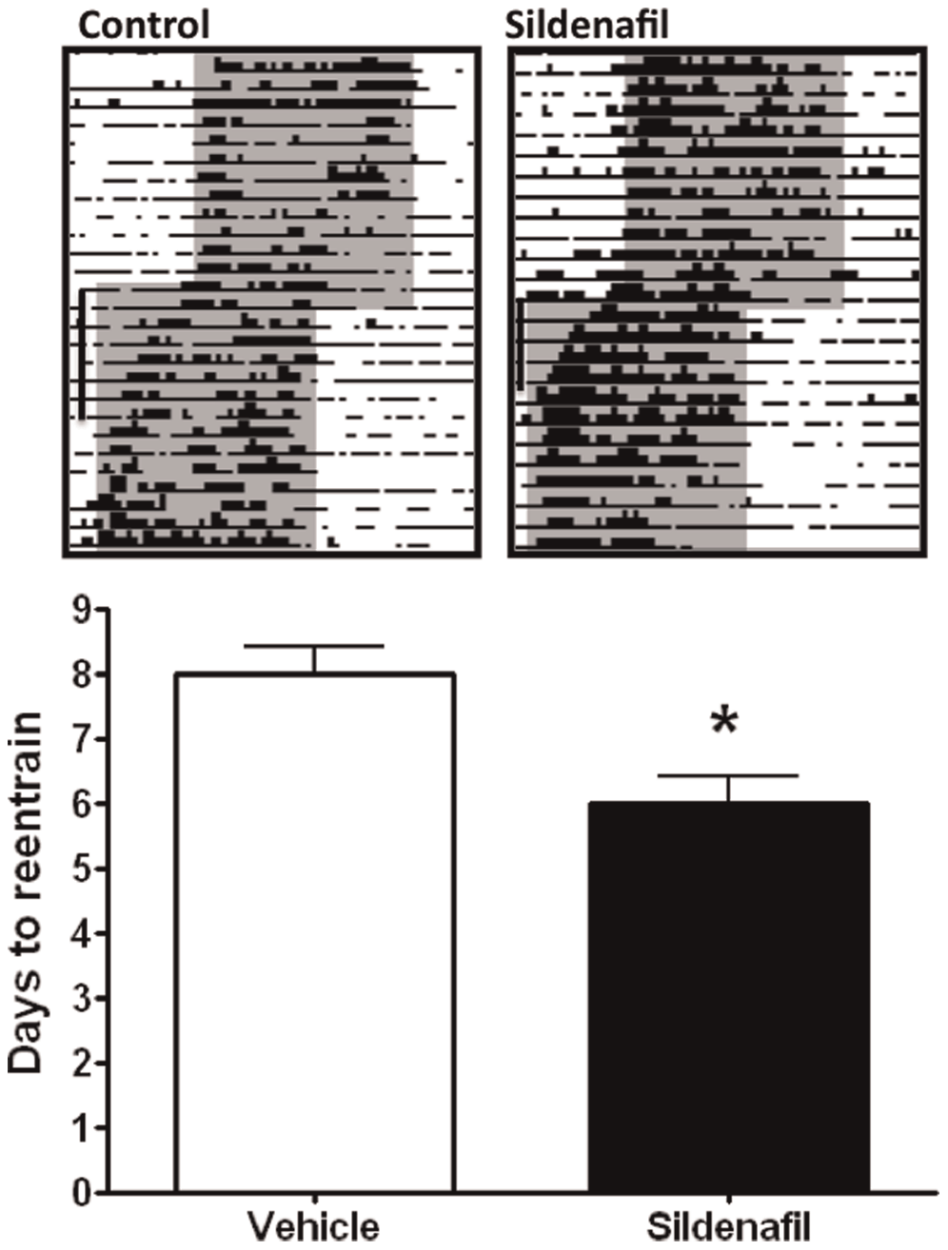
PDE inhibition accelerates circadian reentrainment of body temperature in a jetlag simulation model. Male hamsters injected with sildenafil entrained their body temperature rhythm to a 6-hr advance of the LD cycle significantly faster than those injected with vehicle. N = 6 per group. *p<0.05, Student's t-test. Data represented as mean (±SD). Representative actograms (top) shows body temperature values between 36 and 39°C. Dark phases are indicated by gray shading.

Sildenafil administration also accelerated the reentrainment rate of body temperature in our jet-lag simulation model (Fig. 2). After a 6-h advance of the LD cycle, animals injected with sildenafil (3.5 mg/kg) reentrained their body temperature rhythm significantly faster (6±0.41 days) than those injected with vehicle (8±0.41 days, p<0.05 Student's t-test) (n = 6 per group).

To assess the potential use of orally administered PDE inhibitors to enhance circadian phase advances, male hamsters received 3.5 mg/kg sildenafil through a gavage syringe 30 min before a 300 lux LP at CT18. Oral sildenafil significantly increased the phase-advancing effect of the stimulus (sildenafil 357.3±51.8 min vs. vehicle 123±9.6 min, p<0.025, Student's t-test; n = 6 per group) (Data not shown).

**Figure 3:**
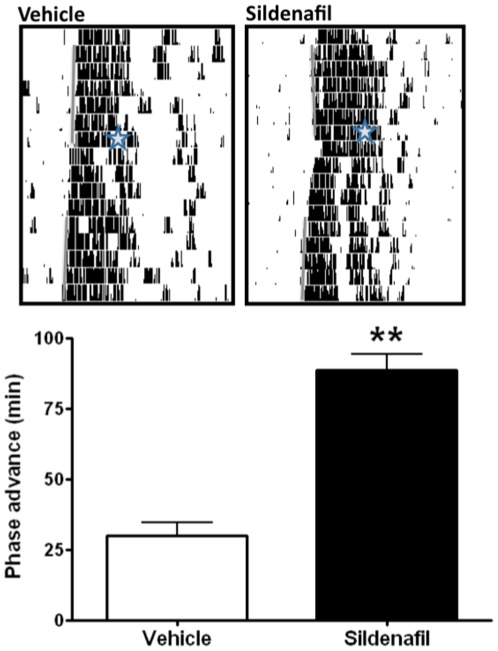
PDE inhibition increases 300 lux light-induced phase advance in mice. 300 lux light pulse (10 min.) at CT19 after sildenafil injection induced a phase advance that was significantly higher than the one for vehicle-treated animals. **p<0,0025 Student's t-test (n = 8 per group). Data represented as mean (±SD) Representative actograms (top) show vehicle or drug treatment, time of injection and LP are indicated by a star.

In order to test if the effects of cGMP manipulation can be generalized to another mammalian species we also studied the photic response in mice, by assessing the effect of light pulses as a proof of concept that sildenafil can affect circadian entrainment in this rodent. The C57-BL/6J mouse strain is a particularly interesting model because it exhibits relatively small phase advances in response to a LP during the late subjective night [19]. As shown in Fig. 3, ip administration of sildenafil induced a 3-fold potentiation of this strain-specific phase-advancing ability: while control mice receiving vehicle 30 min before a 300-lux LP starting at ZT19 showed a phase advance of 30±4.5 min, drug-treated animals (3.5 mg/kg) showed a 88.7±5.06 min phase shift (p<0.0025, Student's t-test; n = 8 per group).

**Figure 4:**
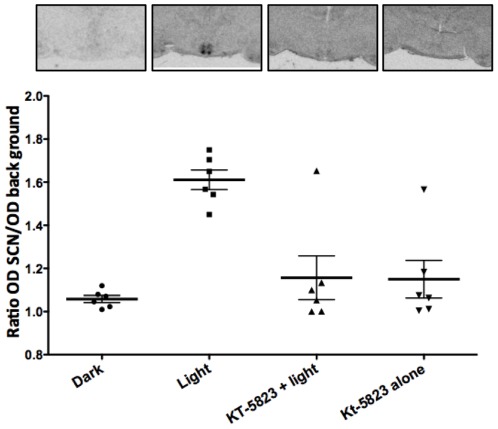
The inhibition of PKG blocks light-induced *Per1* expression in the SCN. Administration of KT-5823 (icv, 200 µM, 1,5 µl), a PKG inhibitor, significantly blocked light-induced *Per1* expression in the hamster SCN (p = 0.023 compared with light, ANOVA followed by Tukey's test), while KT-5823 alone had no effect on *Per1* induction. Data represented as mean of relative OD (ratio OD SCN/OD surrounding hypothalamus) ±SD.

Inhibition of PDE activity after saturating LPs in hamsters at CT 18 and at CT19 in mice (a time when, according to the phase response curve, is not the zone for maximum light-induced phase advances) led to phase shifts that are not encountered under normal conditions in either species. Taken together, these results suggest that the inhibition of cGMP degradation may lead to parameter changes in the master circadian oscillator itself. Phase shifts in mammals are correlated with (and possibly mediated by) the photic-induction of *Per1*, a central gene in the molecular clock machinery. Accordingly, LPs at CT18 induce the acute expression of *Per1* in the SCN (Fig. 4). In order to test whether cGMP levels, and its downstream mechanisms, could directly modulate the increased expression of this core molecular clock component, we administered KT-5823 (i.c.v. 200 µM, 1,5 µl), a PKG inhibitor, which significantly blocked light-induced *Per1* expression. One-way ANOVA (p = 0.023, n = 6 per group) followed by Tukey's test yielded differences between vehicle-treated animals under light or dark conditions (p = 0.0032) without affecting the normal distribution of *Per1* within the SCN. Indeed, an analysis of the signal intensity in the dorsal region vs. the ventral region showeds no difference between the vehicle- and KT-5823-treated hamsters (data not shown). Moreover, PKG inhibition significantly blocked *Per1* induction (light-treated vs. light+KT-5823-treated animals, p = 0.0235) while KT-5823 alone had no effect on *Per* 1 induction (Dark vs KT-5823 alone p = 0.2121).

**Figure 5:**
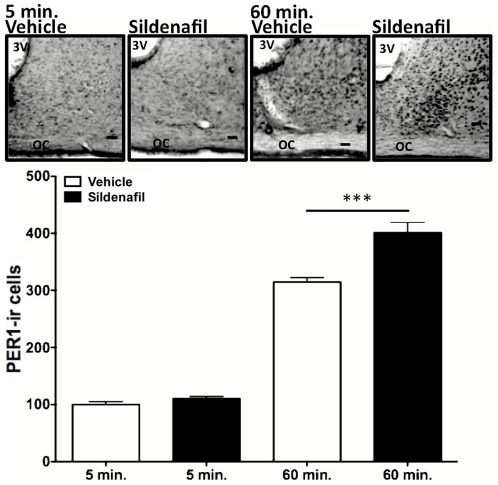
Administration of Sildenafil (3.5 mg/kg) 30 min. before a 300 lux light pulse increase the light-induced phase advance. Animals were sacrificed 5 or 60 minutes after LP. Sildenafil administration increased the number of PER1-ir cells in the SCN. Two-way ANOVA indicated a significant effect of treatment (p<0.0001) and time (p<0.0001), with interaction between these two factors (p = 0.0011) due to the basal expression at 5 min group. A t-Test demonstrates no differences in 5 min group between vehicle an sildenafil (100±4.918 vs. 110±3.96. respectably. p = 0.1294) but shows an increase in the PER1-ir cells at 60 min. (314.4±8.103 vs. 401.1±17.99 respectably. P = 0.0009) (n = 8 per group). Representative coronal sections from hamsters containing the medio-caudal portion of the SCN 5 and 60 min after light pulse with previous injection of sildenafil or vehicle (3 V: third ventricle; OC: optic chiasm; Scale bars: 100 mm).

To better understand how PDE inhibition acts on the circadian clock we analyzed the expression of PER1 protein in the SCN at a short time after the LP. Sildenafil administration increased the number of PER1-ir cells as early as 60 minutes after the LP (Fig. 5). Two-way ANOVA indicated a significant effect of treatment (p<0.0001) as well as of time (p<0.0001), with interaction between these two factors (p = 0.0011). There were no significant differences between vehicle- and sildenafil-treated animals 5 min after the light pulse (100±4.9 min vs. 110±3.9 min, respectively, p = 0.1294, Tukey's test); however, there was significant increase in PER1-ir cells 60 min post-stimulation (control: 314.4±8.1 vs. sildenafil 401.1±17.9 min, P<0.0001, Tukey's test) (n = 8 per group). An analysis of the percentage of PER1-ir cells within the dorsal and ventral regions of the SCN indicated no effect on the distribution of PER1 in these regions when comparing control- and sildenafil-treated hamsters (data not shown).

**Figure 6:**
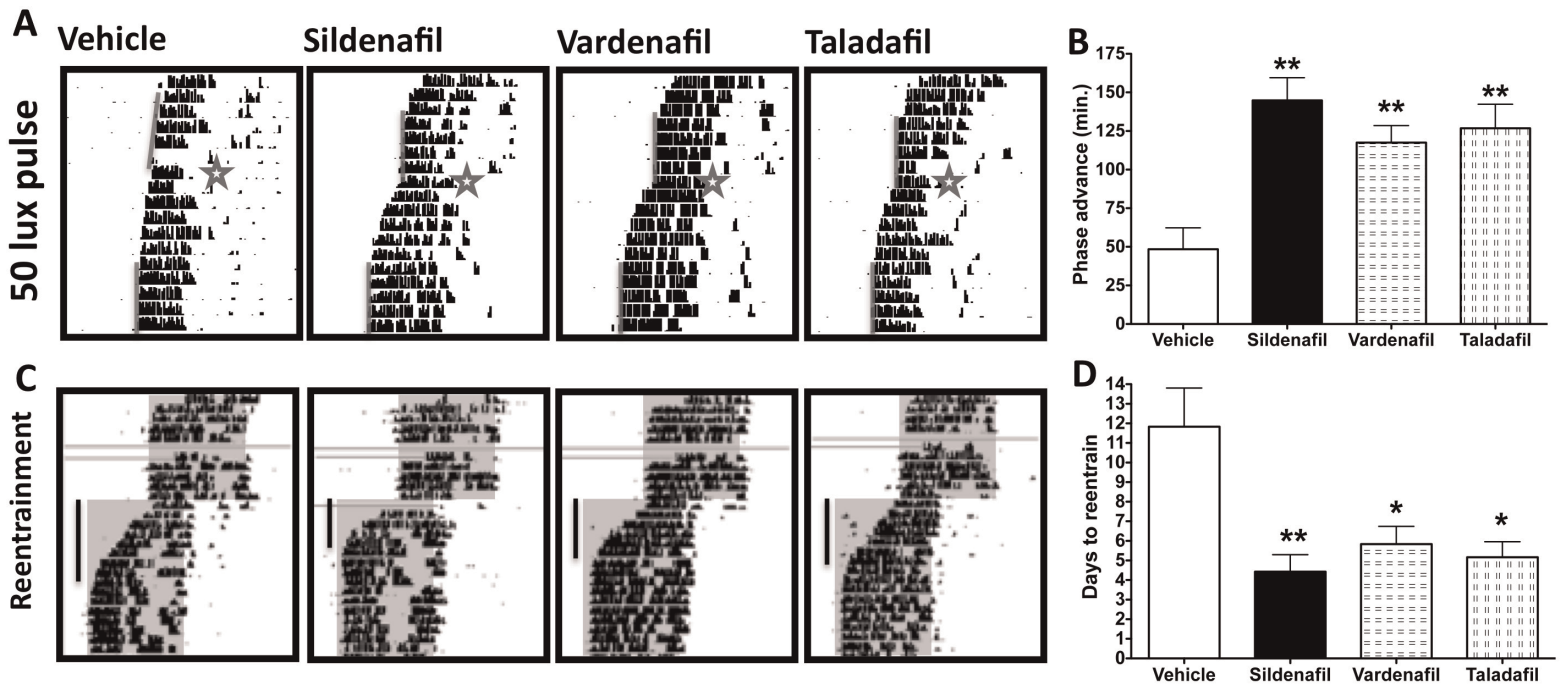
PDE inhibition by different compounds increases low-intensity light-induced phase advances and accelerates circadian reentrainment. A) and B) 50 lux light pulses at CT18 induced a phase advance that was significantly increased by sildenafil, vardenafil or taladafil as compared to vehicle-treated animals. Representative actograms in panel A show vehicle or drug treated-animals; time of injection and LP are indicated by a star and the grey bar shows locomotor activity onsets. Panel B shows the average results, ANOVA-p<0.001- followed by Tukey's Test **p<0,0025. C) Representative actograms showing reentrainment to a 6 h phase advance of the LD cycle for vehicle or drug-treated animals; dark phases are indicated by gray shading and a black bar depicts the transient days until the new phase. Grey horizontal bars indicate computer malfunction. D) Animals injected with PDEs inhibitors entrained significantly faster to a 6-hr advance of the LD cycle than those injected with vehicle. N = 6 per group. ANOVA-P = 0.0011- followed by Tukey's Test *P<0.05, **p<0.0025. All data represented as mean (± SD).

We also studied the effect of several PDE inhibitors on circadian photic responses to subsaturating-LP. The ip. administration of PDE inhibitors sildenafil (3.5 mg/kg), vardenafil (0.7 mg/kg) and tadalafil (1.4 mg/kg) 30 min before a 10-min 50-lux LP at CT18 significantly increased light-induced phase advances as compared to control animals receiving vehicle solution (Fig. 6A, B). The increase in phase shifts was of 290% (sildenafil), 235% (vardenafil) and 252% (Tadalafil) (ANOVA, p = 0.001, followed by Tukey's test of each drug vs. the vehicle, p<0.0025; n = 6 per group).

We also studied the effect of sildenafil, vardenafil and tadalafil on circadian reentrainment after a 6-h advance of the LD cycle (Fig. 6C,D). By recording the time until the start of locomotor activity coincides with the new time for dark onset, we found that control hamsters receiving vehicle synchronized to the new phase in 11.8±1.97 days. Reentrainment time was reduced by half by the ip administration of sildenafil (4.4±0.86 days), vardenafil (5.8±0.91 days) or tadalafil (5.8±0.79 days) (ANOVA, p = 0.0011 followed by Tukey's test for each drug vs. the vehicle, p<0.0025, p<0.05, p<0.05 respectively, n = 6 per group).

In addition, we tested whether there are sexual differences in the ability of PDE inhibitors to increase circadian phase advances. Male or female hamsters receiving an ip. injection of sildenafil 30 min before a 300 lux LP at CT18 exhibited a circadian phase advance of 336±67 min and 349±73 min, respectively, while vehicle-treated animals showed a 204±22 min (males) or 200±37 min (females) phase advance (data not shown). Two-way ANOVA revealed a main effect of treatment (P = 0.0012) but not of sex (P = 0.865; n = 6 per group), suggesting that sildenafil has no sex-specific effect in the potentiation of circadian photoresponsiveness.

## Discussion

The NO-cGMP-PKG pathway is known to be involved in circadian synchronization, more specifically in phase advances of the circadian system in response to light [10], [14], [17], [20]–[23]. cGMP levels increase significantly during the late night after a phase-advancing LP, but do not change during early night photic stimulation, supporting its role in mediating phase advances but not phase delays [12], [24]. In addition, cGMP levels in the hamster SCN exhibit a circadian rhythm with a maximum during the day. This rhythm seems to be related to degradation by a specific PDE, and not to synthesis mediated by GC activity [24]. Thus, the regulation of PDE activity is at the core of the mechanisms underlying the phase-specific effects of light on the circadian pacemaker. In the present work we studied the role of cGMP in the photic response of the circadian system by specific inhibition of PDE5 by sildenafil, tadalafil or vardenafil, three drugs commonly used for treatment of erectile dysfunction [25].

Importantly, sildenafil increased the response not only to a 300 lux LP, but also to a 1200 lux LP (well above saturating threshold). In the latter case, animals exhibited a two-fold increase in phase advances and a significant reduction in the transient days to the new phase. Moreover, the induced increase in phase shifts after 1200 lux pulses (which resulted in ∼9 h phase advances) suggests that sildenafil (and related compounds) increases the photic sensitivity of the circadian clock. Indeed, PDE activity in the SCN might act as an endogenous attenuating factor for light-induced phase advances and its pharmacological inhibition may release the circadian pacemaker from this attenuating effect.

*Per1* is an essential player in the molecular feedback loop that generates circadian rhythms, and is critically involved in resetting the endogenous clock to light signals. *Per1* mRNA and protein levels increase after a LP at late night [26]–[28]. Here we show that PKG inhibition by KT-5823 blocks the light-induced *Per1* expression in the hamster SCN. In addition, our results show that increases in cGMP levels, by means of sildenafil administration, induces a higher number – as compared to controls – of PER1-ir cells in the SCN 60 min after a LP. This increase is concomitant with an enhancement of behavioral phase advances and is consistent with previous reports showing that *Per1* levels in the SCN increase proportionally with light intensity and in parallel with the magnitude of behavioral phase shifts [29]. Our data also support the notion that the phase resetting of the circadian clock likely involves not only the rapid *Per1* activation at subcellular level, but also the recruitment of neuronal oscillators in the SCN [30]. In this way, photic stimulation of the cGMP pathway may not only lead to changes at single-cell level but also in changes in the SCN neuronal network properties.

There are several indications about a possible role of PDE inhibitors for the treatment of central nervous system diseases (i.e., [31]–[33]. In the present study we show that the use of any of three different PDE inhibitors induced a more than two-fold increase in the low-intensity light-induced phase advance, and clearly accelerated behavioral re-entrainment after a 6-h advance in the LD cycle. All three drugs have similar efficacy in both low-intensity light- induced phase advances and re-entrainment after a 6-h advance in the LD cycle. However, although these drugs are clearly specific for PDE5 inhibition, their effect on other isoforms cannot be discarded; for example, tadalafil has been reported as a PDE11 inhibitor but with an IC50 seven times higher than for PDE5 (10 nM and 73 nM respectively) [34]. Several PDE isoforms, in addition to PDE5, are expressed in the SCN according to PCR analysis (Agostino et al., unpublished), and could represent additional targets for pharmacological manipulation of circadian responses to light.

There is a close relationship between the body temperature rhythm and the circadian rhythm of locomotor activity, which suggests a common regulation for the thermoregulatory system and the general sleep pattern [35], [36]. Locomotor activity rhythm has an important influence on core body temperature mainly during the day (when both temperature and activity are low) than during the night [37]. Indeed, resynchronization of body temperature cycles after jet-lag is essential for the restoration of body synchrony and an adequate sleep-wake pattern [38], [39]. In addition, body temperature was recently proposed to be a key component for the internal synchronization of peripheral clocks [40]. Here we show that sildenafil administration also accelerated resynchronization of the body temperature circadian rhythm after a 6-h phase change in the LD cycle. The observed shifts in the body temperature rhythm could in part reflect the effect of locomotor activity as a heat-inducing factor, which can mask the true circadian phase of the temperature rhythm [41], [42]. It is important to note, however, that the effect of PDE inhibition on the reentrainment of the temperature rhythm was observed in hamsters without access to running wheels, in which the effects of heat-induced activity should be less prominent [43], [44]. Our data supports the idea that core body temperature rhythms and locomotor activity rhythms synchronize with different speed to a 6 h advance of the LD cycle. This difference in the speed of reentrainment among different variables was reported previously [45], [46]. The systems that control locomotor activity and temperature rhythms may process the SCN signals in a different way and could be responsible for the differential adjustment rate of output rhythms [47], [48]

In recent years the use of sildenafil has become the therapy of choice for the treatment of erectile dysfunction. Its additional applications in human health, including its use as a chronobiotic drug, remain to be determined, although there have been some indications regarding its efficacy in some sleep-related disorders such as obstructive sleep apnea [49], [50]. In order to further validate the use of sildenafil as a chronobiotic we extended our results to a mouse model. Remarkably, sildenafil was able to enhance light-induced phase advances during the late subjective night in a mouse strain in which this response is very low.

In addition, the use of sildenafil for purposes other than treatment of erectile dysfunction, including in urology and cardiovascular medicine as well as in respiratory dysfunctions has been recommended for males and females (Konstantinos and Petros, 2009). Our results indicate this may be the case for its use as a chronobiotic as well, since it was equally effective in enhancing light-induced phase advances in females as it was in males. Finally, oral administration of sildenafil also proved to be effective in the potentiation of circadian photoresponsiveness, underscoring its potential as an easily deliverable chronobiotic.

Although it is possible that sildenafil might increase retinal sensitivity to light [51]–[54], we have performed experiments that indicate that ip. administration of sildenafil does not change electroretinographic recordings, which, together with the fact that peripheral application of sildenafil results in significant changes in SCN cGMP levels [17], suggests that the results reported herein represent a change at the SCN level. Although other studies have reported that either in vitro or prolongues high doses of sildenafil changes the amplitude of both a and b ERG waveforms [55], [56], the current dose employed in hamsters did not affect retinal electrophysiology.

cGMP effects on photic shifting of the circadian system are mediated through PKG activation [24], [57], since inhibition of this kinase blocks the light-induced phase advance. Moreover, targeted disruption of PKG affects photic entrainment and *Per1-2* expression in the SCN [58]. It is not known how this activation of PKG acts in the circadian system, although some potential substrates have been postulated (e.g., DARPP-32 [59] leading to the activation of clock genes.

Our studies indicate that the cGMP-related signal transduction pathway is a potential pharmacological target for circadian disruptions. In particular, jet-lag as well as shift-work schedules increase the risk of accidents and health problems, including decreased alertness, reduced psychomotor coordination, cognitive deficits, insomnia, reduced performance, loss of appetite, gastrointestinal disease, and even accelerated malignant growth [60]–[62]. Interestingly, several lines of evidence suggest that the response to phase-advancing —as compared to phase-delaying— schedules appears to be more disadvantageous for health and quality of life both in humans and experimental animals [63]–[66]. This outcome may result from an alteration of the internal phase coordination, as well as in the lack of synchrony between the body clock and the environment. Our results indicate that PDE inhibition in conjunction with light administration might be a useful therapy to induce the rapid adaptation of the circadian system to temporal environmental challenges such as rotating shift-work and jetlag.

Genetic sleep disorders may represent another target for PDE-inhibition therapy. For instance, in advanced sleep-phase syndrome, patients report daytime somnolence and difficulties to sleep at night [67], [68]. This syndrome is associated with a specific mutation in the human clock genes *Per2* and CKI delta [69], [70], and current treatments include phototherapy and the use of chronobiotics such as melatonin (or its analogs ramelteon and tasimelteon) or armodafinil [71]–[73]. Our results with experimental animals suggest that PDE inhibition might be added to the list of potentially useful treatments for this and other circadian disorders.

## Materials and Methods

### Ethics Statement

All animal procedures were performed in strict accordance with National Institutes of Health rules for animal care and maintenance. The study protocols were approved by the Universidad Nacional de Quilmes Institutional Animal Care Committee.

### Animals

Male and female adult (3–4 months old) Syrian hamsters (*Mesocricetus auratus*) were raised in our colony and maintained in a 14:10-h LD cycle, with food (Purina chow) and water *ad libitum* and room temperature set at 20±2°C. Adult (2 months old) C57-BL/6J male mice (*Mus musculus*) were raised in our colony under a 12:12-h LD cycle, with food and water *ad libitum* and room temperature set at 20±2°C.

### Locomotor activity Rhythms

Animals were transferred to individual cages equipped with a running wheel (18-cm diameter) and with light intensity averaging 300 lux at cage level. Locomotor activity circadian rhythm was assessed through wheel-running recording (Archron, Argentina). Wheel revolutions were monitored by magnetic microswitches and recorded every 5 min. Time is expressed as Zeitgeber time (ZT), with ZT12 defined as the time of lights off. Animals were initially maintained under a LD cycle for at least 10 days. For reentrainment studies, hamsters were subjected to an abrupt 6-h advance in the phase of the LD cycle, achieved by advancing the time of lights-off and lengthening of the dark phase. On the day of the shift, intraperitoneal (ip.) injection of sildenafil, tadalafil, vardenafil or vehicle was given at ZT18 of the previous cycle. Time for reentrainment to the new LD cycle was defined as the time —expressed in days— it took for each animal to achieve a stable new activity onset in the new cycle. Resynchronization was considered fully accomplished when activity onset took place at the new time of lights off ±15 minutes. Daily onsets of activity were determined following published criteria [47]. Briefly, activity onset was defined as the first 5-min bin that contained at least 80 wheel revolutions, followed by another bin of at least another 80 wheel revolutions within 40 min.

For LP experiments, animals (hamsters or mice) were transferred to individual cages equipped with a running wheel and placed in constant darkness (DD) conditions. For light-induced phase advances of circadian wheel running activity, animals were removed from their cage in DD and exposed to a 10-min white LP of 50, 300 or 1200 lux at circadian time (CT) 18 for hamsters or a 300 lux LP at CT 19 for mice (with CT 12 defined as the onset of wheel running activity in DD). Animals were returned to DD after light exposure. In a previous work we demonstrate that sildenafil administration without light stimulation has no effect in the phase or the period of the animals (Agostino 2007). Phase advances were calculated by fitting a line through activity onsets during the 5 days prior to stimulation and then calculated circadian phase starting on the second day after a stable onset was achieved, using the data of the following 5–7 days to extrapolate to the day of the light pulse. This stable condition was defined as activity onset between consecutive days differing by not more than 15 circadian minutes Phase shifts were estimated by three observers masked to the experimental procedure. Animals received an ip. injection of either drugs or vehicle 30 min before light stimulation and returned into DD. The transient period was defined as the number of days it took for each animal to achieve a new stable phase.

### Drug administration

The effect of 3.5 mg/kg sildenafil, 0.7 mg/kg tadalafil and 1.4 mg/kg vardenafil (extracted from commercial preparations, diluted in 50% DMSO/saline solution) was tested in comparison to vehicle administration [74]. In each experiment, 6 previously untreated hamsters were used for each dosage group or vehicle control. 50% DMSO/saline solution was used as a vehicle.

For the oral administration experiment, 3.5 mg/kg of sildenafil powder (suspended in water) or vehicle were given to hamsters with the assistance of a gavage syringe 30 min before a 300-lux LP at CT18.

### Electroretinography

Electroretinographic activity was assessed in dark-adapted rodents as previously described [75], [76]. Briefly, after 6 h of dark adaptation, male hamsters were injected with 3.5 mg/kg of sildenadil ip, and 30 minutes later were anesthetized with an intraperitoneal injection of ketamine hydrochloride and xylazine hydrochloride under dim red illumination. Phenylephrine hydrochloride (2.5%) and 1% tropicamide (Alcon Laboratories, Argentina) were used to dilate the pupils, and the cornea was intermittently irrigated with balanced salt solution (Alcon Laboratories, Argentina) to maintain the baseline recording and to prevent exposure keratopathy. All recordings were completed within 20 min of the induction of anesthesia. Electroretinograms (ERGs) were recorded from both eyes simultaneously and ten responses to flashes of unattenuated white light (5 ms, 0.2 Hz) from a photic stimulator (light-emitting diodes) set at maximum brightness (9 cd s/m2 without a filter) by a full-field Gandzfeld, were amplified, filtered (1.5-Hz low-pass filter, 1000 high-pass filter, notch activated) and averaged (Akonic BIO-PC, Akonic, Argentina). The a-wave was measured as the difference in amplitude between the recording at onset and the trough of the negative deflection and the b-wave amplitude was measured from the trough of the a-wave to the peak of the b-wave. Electrophysiological responses were averaged for each run. Runs were repeated 3 times with 5 min-intervals, and the mean of these 3 runs was used for subsequent analysis. We compared the mean peak latencies and peak-to-peak amplitudes of the responses from each group of hamsters.

### Body temperature rhythms

To continuously record the body temperature an I-button device (DS1921h-F50, Thermochron, Sunny vale, Ca), set to start 7 days post-surgery, was implanted in the peritoneal cavity of hamsters [77], [78]under 75 mg/kg ketamine and 10 mg/kg xylazine anesthesia cocktail by ip. route. Hamsters were placed in individual cages without running wheels, and body temperature recordings taken every 20 min. Body temperature was filtered to analyze only between 36 and 39°C. Daily onset was defined as the first 5 min-bin with recordings of least 37°C, followed by 8 bins of at least another recording of 37°C within 60 min. Animals were initially maintained under a 14:10-h LD cycle (lights on at 1900 hours) for at least 10 days. Then, hamsters were subjected to an abrupt 6-h advance in the phase of the LD cycle as described above. On the day of the shift, intraperitoneal (ip.) injection of sildenafil or vehicle was given at ZT18 of the previous cycle. Time for reentrainment to the new LD cycle was defined as the number of days it took for each animal to achieve a stable new onset of the temperature rhythm. Resynchronization was considered fully accomplished when body temperature had a new stable phase with a 24 h period.

### PER1 immunohistochemistry

Free-running male hamsters were divided into groups receiving ip. injection of sildenafil or vehicle solution (n = 8 per group) 40 min before a LP (300 lux, 10 min) at circadian time 18 (CT 18). Hamsters were anesthetized with ketamine/xylazine and perfused with 0.1 M phosphate buffer followed by 4% paraformaldehyde in 0.1 M phosphate buffer 5and 60 minutes post LP, and their brains were removed and postfixed for 5 additional hours. The tissue was cryoprotected in 30% sucrose in phosphate buffer saline (PBS) solution and cut into 30 µm coronal sections with a freezing microtome to perform a standard immunohistochemical procedure. Briefly, sections were incubated for 72 h in PBS with 0.4% Triton (PBST) at 4°C with rabbit anti-PER1 antibody (Affinity BioReagents, Rockford, IL, USA) diluted 1∶200, washed in PBST, incubated with biotinylated universal secondary antibody (Vector Labs, Burlingame, CA, USA) diluted 1∶200, and finally incubated with the avidin-biotin complex (Vectastain Elite ABC kit, Vector Labs). The sections were then reacted with Vector VIP reagent (Vector Labs) to visualize the peroxidase reaction, and mounted on microscope slides. The number of PER1 immunoreactive SCN cells (PER1-ir cells) was determined manually using the cell counter plugin of the ImageJ program (NIH). The percentage of ir-cells in the dorsal and ventral regions of the SCN was measured as previously described [21]

### In-situ hybridization of *Per1*

Male Hamsters were sacrificed by decapitation 1 h post LP, brains were rapidly removed and frozen in 2-methylbutane cooled to −30°C with dry ice, and 16-µm thick coronal sections through the SCN were cut on a cryostat and mounted on slides coated with Vectabond (Vector Labs). Sections were processed for in situ hybridization as previously described [79]. Briefly, sections were fixed in 4% paraformaldehyde in 0.1 M phosphate buffer, treated with 0.1 M triethanolamine hydrochloride (pH 8.0) with 0.25% aceticanhydride, rinsed in 2× sodium citrate/sodium chloride (SSC), dehydrated with alcohol and delipidated with chloroform. Sections were incubated in pre-hybridization buffer and hybridized overnight at 55°C in hybridization buffer containing 10% dextran sulfate, 50% formamide, 0.3 M NaCl, 10 mM Tris, 1 mM EDTA, 1× Denhardt's solution, 200 mM dithiothreitol and 0.5 mg/ml yeast tRNA containing4×10^7^ c.p.m./ml of the ^35^S-labeled cRNA probe. The sections were then washed in 50% formamide/50% 2× SSC several times, incubated in RNase A (20 mg/ml) and washed again in formamide/2× SSC. Slides were dehydrated in alcohol, dried and exposed to Hyperfilm-bmax (Amersham, Arlington Heights, IL). All solutions before RNase treatment were prepared with diethyl pyrocarbonate-treated RNase-free water.

The optical density (OD) of the autoradiographic hybridization signal was measured using a Zeiss (Kontron) Image Processing System. The average OD for each SCN was derived from at least two sections through the nucleus, each expressed as a relative OD (ratio OD SCN/OD surrounding hypothalamus).

## Supporting Information

Figure S1 **Electroretinogram of sildenafil treated hamsters.** Sildenafil administration (3.5 mg/kg) does not affect retinal sensitivity to light. Animals were treated with sildenafil or vehicle 30 minutes before ERG, no significant changes were found for amplitude or latency of the *a* or the *b* wave of the ERG after sildenafil administration.
(TIFF)
