# Supplementary figures and images for: cGMP-Phosphodiesterase Inhibition Enhances Photic Responses and Synchronization of the Biological Circadian Clock in Rodents
Source: PLoS One. 2012 May 10;7(5):e37121. doi: 10.1371/journal.pone.0037121 (PMC3349644; doi:10.1371/journal.pone.0037121)

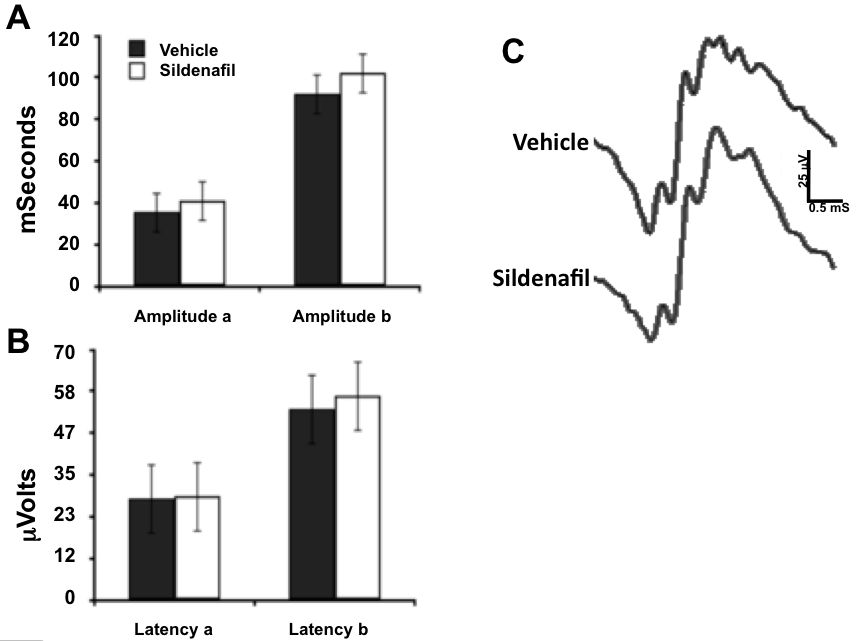

Supplement: Figure S1 — Electroretinogram of sildenafil treated hamsters. Sildenafil administration (3.5 mg/kg) does not affect retinal sensitivity to light. Animals were treated with sildenafil or vehicle 30 minutes before ERG, no significant changes were found for amplitude or latency of the a or the b wave of the ERG after sildenafil administration. (TIFF) [file pone.0037121.s001.tiff]
